# Peer support-based online education, burden of care and quality of life among family caregivers of patients with leukaemia: non-randomised clinical trial

**DOI:** 10.1136/spcare-2023-004610

**Published:** 2024-01-25

**Authors:** Mehrnaz Keramatikerman, Shokoh Varaei, Mohammad Vaezi, Leila Sayadi

**Affiliations:** 1 School of Nursing and Midwifery, Tehran University of Medical Sciences, Tehran, Iran; 2 Department of Internal Medicine, Tehran University of Medical Sciences, Tehran, Iran; 3 School of Medicine, Tehran University of Medical Sciences, Tehran, Iran; 4 Hematology, Oncology and Stem Cell Transplantation Research Center, Tehran University of Medical Sciences, Tehran, Iran; 5 Research Institute for Oncology, Hematology and Cell Therapy Shariati Hospital, Tehran University of Medical Sciences, Tehran, Iran; 6 Nursing and Midwifery Care Research Center, School of Nursing and Midwifery, Tehran University of Medical Sciences, Tehran, Iran

**Keywords:** Leukaemia, Family management, Quality of life, Supportive care, Advance Care Planning

## Abstract

**Objective:**

The responsibility of caring for patients with leukaemia places a heavy burden on family caregivers (FCs) and negatively impacts their quality of life (QoL). This study aimed to investigate the effects of peer support (PS)-based online education programme on the burden of care (BoC) and QoL of FCs of patients with leukaemia.

**Methods:**

This before-after study involved a total of 80 eligible FCs of patients with leukaemia (40 individuals per group). The participants received the necessary information from a researcher and peers through online sessions and WhatsApp group. To collect data, the Zarit Burden Interview and the Caregiver Quality of Life Index-Cancer (CQOLC) had been been completed once before the intervention and once 1 month after the intervention.

**Results:**

There was no significant difference between the two groups regarding baseline variables except the mean BoC that was significantly higher in the intervention group (IG) (p<0.001). However, after controlling for the effects of confounding variables, the mean BoC score of participants in IG was significantly lower than that of the control group (p<0.001). Additionally, there was no significant difference between the two groups in terms of CQOLC before (p=0.178) and after (p=0.538) the intervention.

**Conclusion:**

The PS-based online education programme had a positive impact on reducing the care burden of FCs of patients with leukaemia. This programme can effectively reduce costs, particularly during emergencies and crises such as pandemics, as it eliminates the need for FCs and peers to physically visit hospitals.

**Registration:**

The study was registered at the Iranian Registry of Clinical Trials on 18 July 2021 (IRCT registration number: IRCT20210507051209N1).

**WHAT IS ALREADY KNOWN ON THIS TOPIC**

- FCs of patients with leukemia sufferers bear a caregiving burden.

**WHAT THIS STUDY ADDS**

- PS-based online education for FCs of patients with leukaemia may reduce their BoC.

**HOW THIS STUDY MIGHT AFFECT RESEARCH, PRACTICE OR POLICY**

- Not needing the participation of FCs and peers in person can enable the use of PS-based online education in hospitals for FCs of patients with leukaemia. More research on FCs of patients with other cancer can clarify the application of this type of education.

## Background

 Leukaemia is a malignancy of the haematopoietic system, which is classified into myeloid and lymphoid types based on the characteristics of the affected cells and into acute and chronic forms according to the speed of proliferation.^1^ Chemotherapy and haematopoietic stem cell transplantation (HSCT) are common treatments for leukaemia patients, but they are associated with several physical complications and psychological problems.^2^ Despite significant advances in the treatment of leukaemia, problems such as the high number of patients and the shortage of hospital beds have shifted the focus of care from hospitals to homes by family caregivers (FCs).^3^

FCs of patients with leukaemia spend a great deal of time from the time of diagnosis through treatment performing nursing and medical tasks and helping patients with their daily activities.^3 4^ Therefore, the heavy workload and responsibility combined with the lack of preparation put tremendous pressure on caregivers.^3 4^ FCs of patients with cancer also face many economic, psychological and physical problems.^3^ Because of the sudden diagnosis, the high cost and the vital decisions that must be made immediately after diagnosis, the burden of care (BoC) placed on FCs of patients with leukaemia is much higher than that assumed by caregivers of patients with other chronic and progressive diseases.^5 6^

BoC that FCs face significantly reduces their quality of life (QoL).^5 7 8^ An integrative review to identify factors affecting cancer caregiver burden and QoL found a moderate negative correlation between burden and QoL.^9^ A concept analysis also showed that caregiver burden leads to negative changes, including reduced care provision and lower QoL.^10^ A study conducted in Turkey found that families of patients with leukaemia who undergo peripheral HSCT experience an association between their QoL and BoC.^5^ It is noteworthy that providing care for patients with cancer causes extreme psychological distress for FCs and can turn them into patients themselves.^11^

Several researchers have attempted to decrease BoC and improve the QoL of FCs of patients with cancer through education and counselling programmes,^12 13^ with varying levels of efficacy. Today, interventions are increasingly focused on supportive and social programmes, such as peer support (PS) programmes, offered through social networks. These interventions allow FCs to access valuable resources, including health information, instructions for simple stress relief activities and PS groups (PSGs), via cell phones or personal computers.^14^ Online PSGs enable individuals facing similar situations to easily exchange their views and ideas. Many caregivers find it helpful to exchange health information and share their experiences with peers in these online groups. Studies found that PSG sessions are especially useful for young and educated individuals suffering from severe distress.^15 16^ In particular, in these researches, nurses played a vital role in decreasing family caregiver burden by providing education, support, guidance and resources to FCs. Also, nurses can identify FCs who have successfully provided care for patients and encourage them to support other FCs by PSGs. Nurses can facilitate communication between FCs and PSGs, providing education about the importance of PS and emotional support for FCs participating in PSGs.^17^

The literature review indicates that most previous education or PS interventions, particularly in Iran, have involved face-to-face sessions.^18^ However, due to the emergence of COVID-19 and the possibility of future pandemics, researchers in recent years have to increasingly focus on online interventions. FCs can use online programmes from the comfort of their own homes whenever they want.^19^ To our knowledge, no previous research has investigated the effects of education or PS-based interventions on BoC for FCs of patients with leukaemia in Iran. Therefore, this study aimed to investigate the effects of a PS-based online education programme on BoC and QoL of FCs of patients with leukaemia.

## Methods

### Trial design

This non-randomised clinical trial was conducted using before-after design from 22 December 2021 to 5 June 2022.

### Participants and setting

A total of 80 individuals over 18 years of age who were primary FCs of patients with leukaemia and willing to participate in the study were enrolled. They had to be able to read and write and had access to the internet and cell phone. These FCs were providing care for patients with leukaemia in the 18–60 age group who were candidates for induction or consolidation chemotherapy and hospitalised more than 10 days. The non-inclusion criteria included substance abuse and self-reported mental illness. Also, FCs who experienced a stressful life event (eg, separation, death of another family member and simultaneous care of a family member with another illness) were not entered. FCs that the patient for whom they were caring died were excluded. Experiencing a stressful life event during the study was also considered an exclusion criterion, but none of the participants reported this. The criteria were determined by expert panel (EP). EP consisted of researchers, two haematologist and oncologist specialists and two expert head nurses of haematology-oncology wards of Shariati hospital.

The participants were selected among FCs of patients with leukaemia who underwent chemotherapy in the hematology-oncology wards of Shariati hospital, affiliated with Tehran University of Medical Sciences (TUMS). This hospital is one of the main referral centres for HSCT treatment in Iran.

FCs were invited to participate in the study by one of the researchers (MK) who was working in the wards. MK was attending in the hospital and explaining the purpose of the study, inviting FCs to participate in the study. All FCs who met inclusion criteria were invited to participate in the study (n=177).

### Intervention

The intervention consisted of two parts: an education programme conducted by the researchers and peers and a PSG programme. The intervention was designed by EP.

At first, the researcher (MK) consulted with expert head nurses of haematology-oncology wards to select eligible peers. The head nurses worked in haematology-oncology wards for many years and were familiar with patients with leukaemia and their FCs. Ten individuals were selected as peers who had successfully cared for patients with leukaemia as primary FCs in the past, had a high school diploma or higher, were able to communicate effectively and were willing to participate in the study. The EP evaluated the peers, and two female peers and two male peers were selected. Table 1 presents the main characteristics of the peers.

**Table 1:** Characteristics of peers

Peer no.	Age (year)	Gender	Relation with the patient	Type of leukaemia	Time since diagnosis
1	36	Female	Spouse	AML	1 year
2	35	Female	Spouse	ALL	9 months
3	33	Male	Brother	ALL	2 years
4	22	Male	Spouse	AML	1.5 years

ALLacute lymphoblastic leukaemiaAMLacute myeloid leukaemia

In a face-to-face meeting, one of the researchers (MK) explained the research objectives and intervention in detail to the peers, provided a brief description of leukaemia and discussed the available treatment and care options. Then peers were informed that they did not need to come to the hospital to provide support and they would only participate in the study via WhatsApp. They were also advised to only share their caregiving experiences with FCs.

A WhatsApp group was created so that participants could receive PS and necessary information from the researchers and peers. In this group, researchers and all peers were added to provide information and support to FCs. Each FC was also added to this group after checking of eligibility to participate. The researchers held two sessions, 1 day apart, for participants via WhatsApp. During these sessions, the researchers provided FCs with information about different types of leukaemia and care procedures in hospitals and at home using photos, texts, voice messages and video clips. In addition, in these sessions, at least two of the peers contributed by sharing their similar experiences with participants and explaining patient care options. Separate sessions were also held for those who joined the group later.

**Figure 1:**
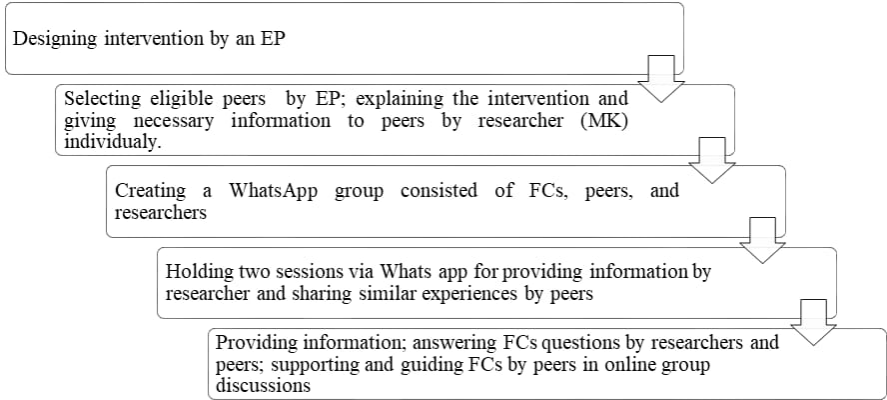
Intervention steps. EP, expert panel; FCs, family caregivers.

Then peers and FCs were asked to attend online sessions in the WhatsApp group 3 days a week, for 4 hours a day, with at least one researcher and one peer present in the group at all times. The meetings took place from 7:00 pm to 11:00 pm to help FCs . In a defined period, the researcher provided participants with necessary educational content and information; also peers used texts or voice messages to share their experiences in providing care for patients with leukaemia. The researcher answered participants’ questions about the disease and the care process, while peers shared their similar experiences with participants. Moreover, peers offered support and guidance to FCs who freely shared their feelings about caregiving and discussed their relevant problems. Peers also provided information to participants about institutions they could go to for help. During this time, only the researchers and peers presented all the time online. FCs were free to ask questions at any time within the allotted time. In other words, FCs did not need to be online for the entire period. In all sessions, one of the researchers (MK) supervise all texts, voices and answering of the questions by peers. The programme continued for a month, even after the patients were discharged from the hospital. Figure 1 provides a summary of the intervention phases.

Members of the control group (CG) received routine care only from nurses and physicians. The routine care, which included some pamphlets and information about the disease and the care process, was also provided to members of the intervention group (IG).

### Outcomes

The study measured BoC and QoL of FCs of patients with leaukamia as primary and secondary outcomes, respectively, using the Zarit Burden Interview (ZBI) and the Caregiver Quality of Life Index-Cancer (CQOLC) scales.

### Data collection

The study collected demographic and clinical information from patients, including age, gender, educational qualifications and type of leukaemia. Additionally, the study collected demographic information from FCs, including age, gender, marital status, educational qualifications, occupation, relationship with the patient, underlying diseases (severe, acute or chronic disease including respiratory system, cardiovascular, metabolic, nervous system and urinary system) and duration of care.

**ZBI**: This 22-item tool was designed by Zarit *et al* to measure BoC. The questions of ZBI include personal, social, emotional and economic pressures. The items are scored on a five-point Likert scale, including ‘never’ (0) to ‘nearly always’ (4). The total score ranges from 0 to 88, with higher scores indicating a greater perceived BoC. In addition, scores from 0 to 20, 21 to 40 and 41 to 88 indicate little or no burden, moderate burden and severe burden, respectively.^20 21^ The Persian version of the ZBI has been used in Iran.^22^ In this study, the reliability of the ZBI was confirmed with a Cronbach’s alpha value of 0.83.

**CQOLC scale**: This questionnaire was originally developed by Weitzner *et al*^23^ to measure the QoL of FCs of patients with cancer. The 35-item scale is scored on a five-point Likert scale that includes ‘not at all’ (0) to ‘very much’ (4). The total score ranges from 0 to 140, with higher scores indicating greater QoL. The four domains of the CQOLC scale include mental/emotional burden (14 items, score range: 0–56), lifestyle disruption (9 items, score range: 0–36), positive adaptation (8 items, score range: 0–32) and financial concerns (3 items, score range: 0–12). An additional item, family interest in caregiving, is not categorised in the above domains but is calculated to measure the total QoL score.^24^ In Iran, the validity and reliability of the Persian version of this scale have been confirmed.^25^ In the present study, the reliability of the tool was confirmed with a Cronbach’s alpha value of 0.72.

The researcher (MK) collected patients’ demographic and clinical information by reviewing their medical records. She also sent the links to the electronic questionnaires to the FCs, who completed the demographic characteristic questionnaire at baseline and the ZBI and CQOLC scales before and 1 month after the intervention.

### Sample size

The sample size was determined to be 35 individuals per group based on the observed changes in BoC variable in IG compared with CG.^12^ The final sample size was calculated as 40 individuals per group, allowing for a 15% loss to follow-up.

### Sampling and randomisation

Due to the limited number of patients with leukaemia at Shariati hospital, eligible participants were selected using convenience sampling. To avoid contamination, the researcher (MK) first selected members of CG and then selected 40 individuals as members of IG.

### Blinding

The participants and the researcher (MK) were not blinded and the statistician was the only person who did not know the allocation sequence.

### Statistical analysis

Continuous and categorical variables were described with mean±SD and frequency (percentage) indices, respectively. Independent sample t-test and paired sample t-test were applied to compare variables between and within groups, respectively. The univariate comparison of postoutcomes (BoC and CQOLC after intervention) was made by the analysis of covariance by adjusting baseline levels of the relevant outcome as a covariate. Also, the association between two groups with demographic categorical characteristics was analysed using χ^2^ test or Fisher exact test. Finally, to compare the effect of the intervention on outcomes with adjusting for confounding factors (patient demographic and clinical data and caregiver demographic) and baseline levels of the relevant outcome, a backward stepwise multiple linear regression model (with entry p=0.05, removal p=0.1 criterion) was used. All statistical analyses were performed using SPSS V.16.0, and a p value of 0.05 was considered statistically significant.

.

## Results

**Figure 2:**
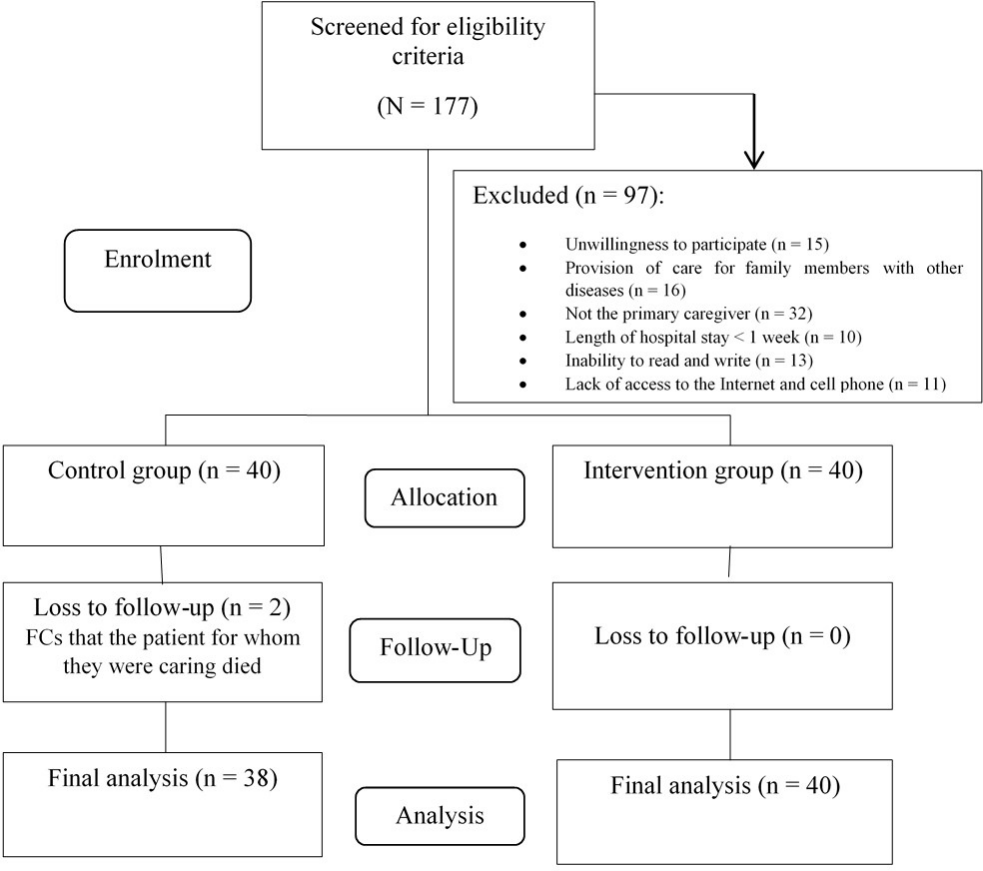
Consort flow diagram of the study. FCs, family caregivers.

As shown in figure 2, a total of 177 FCs were screened for inclusion criteria, of whom 97 were excluded. Finally, 80 eligible participants were assigned to IG and CG, with 40 individuals per group.

### Baseline variables

Acute myeloid leukaemia (AML) was the most common type of leukaemia in both IG (26 individuals, 65%) and CG (23 individuals, 60.5%). Additionally, no significant difference was observed between the intervention (0.760±0.794) and control (0.584±0.437) groups in terms of the mean duration of care (p=0.233). There were no significant differences between the two groups regarding other baseline variables (table 2).

**Table 2:** Baseline demographic and clinical characteristics of patients and FCs

Variables		Patients			FCs		
		Intervention	Control	P value	Intervention	Control	P value
		N (%)Mean±SD	N (%)Mean±SD		N (%)Mean±SD	N (%)Mean±SD	
Age (year)		34.70±9.17	34.97±11.84	0.909	34.35±10.76	36.44±10.84	0.394*
Gender	Male	29 (72.5)	23 (60.5)	0.338	14(35)	14 (36.8)	1†
	Female	11 (27.5)	15 (39.5)		26(65)	24 (63.2)	
Marital status	Single	11 (27.5)	14 (36.8)	0.693*	11 (27.5)	11 (28.9)	0.607†
	Married	25 (62.5)	21 (55.3)		24(60)	25 (65.8)	
	Widowed or divorced	4 (10)	3 (7.9)		5 (12.5)	2 (5.3)	
Educational qualifications	High school diploma and lower	8 (20)	8 (21.1)	0.955	6 (15)	5 (13.2)	0.346†
	High school diploma	15 (37.5)	13 (34.2)		15 (37.5)	9 (23.7)	
	Academic degrees	17 (42.5)	17 (44.7)		19 (47.5)	24 (63.2)	
Type of leukaemia	ALL	14(35)	15 (39.5)	0.815	–	–	–
	AML	26(65)	23 (60.5)		–	–	
Job	Housewife or unemployed	–	–	–	15 (37.5)	14 (36.8)	0.484†
	Self-employed	–	–		15 (37.5)	13 (34.2)	
	Employee	–	–		10(25)	11 (28.9)	
Economic status	Low	–	–	–	6 (15)	4 (10.5)	0.337†
	Moderate	–	–		27 (67.5)	22 (57.9)	
	Good	–	–		7 (17.5)	12 (31.6)	
Relation with the patient	Father/mother	–	–	–	7 (17.5)	8 (21.1)	0.659†
	Brother/sister	–	–		14(35)	12 (31.6)	
	Spouse	–	–		15 (37.5)	11 (28.9)	
	Child	–	–		4 (10)	7 (18.4)	
Underlying disease	Yes	–	–	–	6 (15)	6 (15.8)	0.923†
	No	–	–		34(85)	32 (84.2)	
Duration of care (months)		–	–	–	0.760±0.794	0.584±0.437	0.233*

* iIndependent samples t-test.

† chi-squaredχ2 test ; Fisher’s exact test.

ALLacute lymphoblastic leukaemiaAMLacute myeloid leukaemiaFCsfamily caregivers

### Outcomes

Before the intervention, the mean BoC score of participants in IG (51.80±6.362) was significantly higher than that of those in CG (37.26±13.383) (p<0.001). However, after the intervention, the mean BoC score of participants in IG (47.10±6.037) was significantly lower than that of those in CG (56.13±6.99) (p<0.001). In addition, there were significant differences within IG (p=0.002) and CG (p<0.001) in terms of before and after BoC scores (table 3).

**Table 3:** Comparison of outcomes between intervention group and control group

Primary outcome			Range	Intervention	Control	P value
				N (%)Mean±SD	N (%)Mean±SD	
BoC (before)	Total score		–	51.80±6.362	37.26±13.383	<0.001*
	Level	Little or no burden	–	0 (0)	2 (5.3)	<0.001†
		Moderate burden	–	2 (5)	22 (57.9)	
		Severe burden	–	38 (95)	14 (36.8)	
BoC (after)	Total score		–	47.10±6.037	56.13±6.999	< 0.001‡
	Level	Little or no burden	–	0 (0)	0 (0)	0.055†
		Moderate burden	–	5 (12.5)	0 (0)	
		Severe burden	–	35 (87.5)	38 (100)	
Differences within the two groups in before and after scores (p value)				0.002§	<0.001§	
**Secondary outcome**						
CQOLC (before)	Total score		46–113	68.95±6.84	72.78±16.41	0.178*
	Mental/emotional burden		8–49	26.58±3.65	25.79±9.06	0.621
	Lifestyle disruption		12–33	17.70±3.61	21.74±6.02	0.001
	Positive adaptation		8–29	16.48±3.47	18.34±4.85	0.056
	Financial concerns		0–12	6.20±2.85	4.66±3.38	0.032
CQOLC(after)	Total score		51–98	67.25±7.04	69.47±9.86	0.253‡
	Mental/emotional burden		15–41	26.10±3.99	25.63±6.57	0.703
	Lifestyle disruption		6–24	15.53±3.75	17.74±2.87	0.005
	Positive adaptation		5–26	18.25±3.69	17.76±4.51	0.603
	Financial concerns		1–12	5.88±3.07	6.55±2.85	0.316
Differences within the two groups in before and after scores (p value)				0.216§	0.242§	

* ( Independent samples t-test; .

† Fisher’s exact test; Chi-squareχ2 t-test; .

‡ Analysis of Ccovariance () (considering before outcome as covariate);.

§ Paired sample t-test).

BoCburden of careCQOLCCaregiver Quality of Life Index-Cancer

No significant difference was found between IG and CG in terms of CQOLC before (p=0.178) and after (p=0.253) the intervention. In addition, there was no significant difference within IG (p=0.216) and CG (p=0.242) in terms of before and after CQOLC scores (table 3).

After controlling for the effects of confounding variables, female caregivers and caregivers of patients with ALL were found to experience higher levels of BoC than male caregivers and caregivers of patients with AML. The duration of care was directly associated with BoC. In addition, a significant difference was found between the two groups in terms of the mean BoC score (p<0.001) (table 4).

**Table 4:** Multiple linear regression analysis evaluating the relationship of burden of care and patients’/family caregivers’ characteristics before and after the intervention

Independent variable	Coefficients	SE	95% CI	P value
Group (control vs intervention)	7.711	1.742	4.238 to 11.183	<0.001
Patient’s gender (female vs male)	2.728	1.519	−0.300 to 5.756	0.077
Type of leukaemia (AML vs ALL)	−2.787	1.464	−5.705 to 0.130	0.061
Duration of care	2.478	1.128	0.230 to 4.726	0.031
BoC (before)	−0.090	0.070	−0.229 to 0.049	0.203
R square (R^2^)=0.43*, F (5.72)=0.68, p<0.001				

Dependent Vvariable: BoC (Aafter);, Bbackward Mmethod (keeping the Ccare burden-Bbefore variable in the model).

* The regression model is statistically significant and explains 43% of the observed variability in the dependent variable.

ALLacute lymphoblastic leukaemiaAMLacute myeloid leukaemiaBoCburden of care

After controlling for the effects of confounding variables, it was found that female FCs had a higher CQOLC than that of male caregivers. Caregivers with good economic status also had a better QoL than those with poor financial status. Additionally, patients’ siblings had a higher QoL than patients’ spouses and children. Finally, no significant difference was observed between the two groups in terms of the mean CQOLC score (p=0.538) (table 5).

**Table 5:** Multiple linear regression analysis evaluating the relationship of Caregiver Quality of Life Index-Cancer and patients’/family caregivers’ characteristics before and after the intervention

Independent variable		Coefficients	SE	95% CI	P value
CQOLC (before)		0.144	0.077	−0.010 to 0.299	0.067
Patient’s age		−0.213	0.122	−0.457 to 0.031	0.087
FCs’ gender (female vs male)		4.344	1.933	0.485 to 8.202	0.028
Patient’s marital status	Married	5.050	2.846	−0.632 to 10.732	0.081
	Single, widowed or divorced	7.257	4.254	−1.237 to 15.750	0.093
FCs’ economic status*	Moderate	3.252	2.888	−2.515 to 9.019	0.264
	Good	7.396	3.269	0.868 to 13.923	0.027
FCs’s relation with the patient†	Brother/sister	−5.753	2.863	−11.469 to −0.038	0.049
	Spouse	−5.828	3.453	−12.723 to 1.067	0.096
	Child	−3.073	4.573	−12.204 to 6.058	0.504
Group (control vs intervention)		1.161	1.874	−2.581 to 4.902	0.538
R square (R2)=0.27‡, F (11,66)=2.214, p=0.024)					

* Reference category: poor; .

† Reference category: Pparents:, Ddependent Vvariable: (CQOLC Aafter); Sselection Mmethod:, Bbackward (keeping the CQOLC Bbefore variable in the model).

‡ The model is statistically significant and accounts for 27% of the observed variability in the dependent variable.

CQOLCCaregiver Quality of Life Index-CancerFCsfamily caregivers

## Discussion

The PS-based online education programme reduced BoC for FCs of patients with leukaemia but had no significant impact on FCs’ QoL.

Before the intervention, the mean BoC score of participants in IG was significantly higher than that of those in CG. Data for CG and IG were collected from 22 December 2021 to 11 March 2022 and from April 9 to 5 June 2022, respectively. Since international sanctions and economic problems, such as inflation,^26^ were more severe over time, these problems likely increased patients’ medical costs and thus placed a greater burden on FCs.

After the intervention, the mean BoC score of participants in IG was significantly lower than that of those in CG. Therefore, the intervention effectively reduced BoC on FCs. Only a few researchers have examined peer-support-based online education programmes. On the other hand, studies that have investigated the effects of education programmes and telephone counselling on BoC for FCs have generally yielded conflicting results. In a study in Iran, telephone counselling reduced BoC experienced by cancer patients’ caregivers.^27^ The results of a Taiwanese study showed that providing integrated inperson caregiver support significantly reduces subjective caregiving burden in caregivers of patients with advanced cancer.^28^ Caregivers of patients other than patients with leukaemia have also benefited from the advantages of various interventions. For example, in a study in Turkey (2019), home-based care and caregiver education reduced BoC for caregivers of patients who had stroke.^29^ However, the results of a study in Australia indicated that a telephone outcall programme had no significant effect on BoC for caregivers of patients with cancer.^13^ Similarly, in a systematic review, electronic health-based interventions had no significant impact on BoC for caregivers of patients with cancer.^30^ It should be noted that several social, cultural, individual, family and health factors influence BoC^31^ ; hence, different results may be obtained in different countries. In the present study, the PS-based online programme reduced BoC for FCs during the COVID-19 pandemic.

The PS-based online programme did not have a significant influence on the QoL of FCs. In contrast to this finding, a caregiver education programme significantly improved the QoL of caregivers of patients with cancer in a study in France. The intervention aimed to improve participants’ skills in areas such as nursing, meal support, symptom management and patient care.^12^ In another study, a social support programme and coping strategies significantly enhanced the QoL of caregivers of patients with cancer.^32^ Additionally, the family PS programme was found to positively affect the QoL of FCs.^33 34^ On the other hand, some researchers have concluded that the PS programme does not significantly influence the QoL of caregivers.^33 35^ These studies used different approaches to design and execute the education and PS programmes. Given that different cultural values, beliefs and family systems affect the QoL of individuals,^36^ the designed programme did not significantly improve the QoL of FCs of patients with leukaemia in Iran.

The online nature of the programme made it easy for FCs to receive PS and the information they needed from the researcher and peers. Therefore, considering that most people in Iran nowadays have access to the internet, the online education approach can provide FCs with the necessary training and support. In addition, online education and support approaches have been found to effectively reduce anxiety, increase self-efficacy and meet patient needs.^37 38^ Therefore, online programmes can provide support to FCs and improve the quality of services, especially in emergencies such as the COVID-19 pandemic when caregivers and peers cannot attend inperson meetings. Given the chronic nature of cancer and its high economic and psychological burden on patients, families and society and the fact that FCs provide the majority of healthcare services to these patients, the physical, mental, economic and social well-being of FCs can directly affect the quality of services they provide to patients.^8 39^ The PS-based online programme can effectively reduce BoC for FCs of leukaemia patients. Researchers are encouraged to investigate the effectiveness of the programme in improving patient outcomes and reducing related costs in future studies.

Nurses providing care for patients with leukaemia, as well as those working in inpatient and chemotherapy units, can implement this intervention to provide FCs with the necessary training and information. They can also identify experienced FCs who have successfully provided care for patients with leukaemia and encourage them to support other caregivers. Furthermore, relevant authorities are recommended to emphasise the importance of online education, modern educational technologies and PS in educating and supporting patients and FCs in refresher courses organised for nurses, nursing supervisors and nurse managers.

### Limitations

Shariati Hospital is the main centre in Iran that provides HSCT treatment for adults with leukaemia; therefore, due to the limited size of the study population, the researcher had to use convenience sampling rather than random sampling. Also, IG and CG were not randomly allocated and participated at different time points, so this could have an effect on the results. In addition, due to time constraints and the limited number of patients, the researcher was unable to mitigate sample heterogeneity in terms of age, gender, type of care and so on. Given the limitations of finding peers who would participate in research, the short distance between a family member’s diagnosis and peers’ participation may have placed burden on peers. However, they willingly participated in this study.

## Conclusion

It is encouraging to see that FC’s BoC was reduced through the PS-based online education programme; however, this is unclear because IG and CG were involved at different times and there was no randomisation. Future research directions, including large randomised controlled trials, should be specifically designed. Given that online programmes do not require caregivers and peers to attend inperson meetings, these approaches provide FCs with low-cost and time-efficient training and support, especially during emergencies and crises such as COVID-19 pandemics.

## Data Availability

Data are available upon reasonable request.
